# Modulation of the intermolecular interaction of myoglobin by removal of the heme

**DOI:** 10.1107/S0909049513022772

**Published:** 2013-10-02

**Authors:** Hiroshi Imamura, Takeshi Morita, Tomonari Sumi, Yasuhiro Isogai, Minoru Kato, Keiko Nishikawa

**Affiliations:** a Graduate School of Advanced Integration Science, Chiba University, 1-33 Yayoi, Inage, Chiba 263-8522, Japan; b Department of Chemistry, Okayama University, 3-1-1 Tsushima-Naka, Kita-ku, Okayama 700-8530, Japan; c Department of Biotechnology, Toyama Prefectural University, 5180 Kurokawa, Imizu, Toyama 939-0398, Japan; d Department of Pharmacy, Ritsumeikan University, 1-1-1 Nojihigashi, Kusatsu, Shiga 525-8577, Japan

**Keywords:** small-angle X-ray scattering, protein–protein interaction, protein engineering

## Abstract

Toward understanding intermolecular interactions governing self-association of proteins, the present study investigated a model protein, myoglobin, using a small-angle X-ray scattering technique. It has been known that removal of the heme makes myoglobin aggregation-prone. The interparticle interferences of the holomyoglobin and the apomyoglobin were compared in terms of the structure factor. Analysis of the structure factor using a model potential of Derjaguin–Laudau–Verwey–Overbeek (DLVO) suggests that the intermolecular interaction potential of apomyoglobin is more attractive than that of holomyoglobin at short range from the protein molecule.

## Abstract 2

The present study analysed small-angle X-ray scattering profiles of myoglobin to examine how removal of the heme changes the intermolecular interaction.

## 1. Introduction

Precipitation or aggregation of proteins is often problematic when handling protein solution for research and commercial use. Developing methods to inhibit undesired aggregation of proteins is one of the critical issues in the biopharmaceutical field (Shire *et al.*, 2004 ▶; Yang *et al.*, 2010 ▶). To our knowledge, in protein design studies, we often come across water-insoluble artificial proteins (Isogai *et al.*, 2005 ▶; Imamura *et al.*, 2012 ▶). In contrast, a mechanism which prevents an uncontrolled aggregation is likely to exist in naturally occurring proteins (Isogai, 2006 ▶; Monsellier & Chiti, 2007 ▶). Understanding physicochemical mechanisms governing self-association of proteins will be useful for future protein engineering. In the present study, we used a small-angle X-ray scattering (SAXS) technique to analyze protein’s interparticle interference, which yields an intermolecular interaction of a protein in solution through use of the theory for liquids (Tardieu *et al.*, 1999 ▶). We employed myoglobin as a model protein. Removal of heme is regarded as a kind of simple protein engineering. Interestingly, it was reported that the apomyoglobin, in which the heme is removed, is prone to aggregate, while holomyoglobin, in which the heme is binding, is highly water-soluble (Fändrich *et al.*, 2001 ▶, 2003 ▶). Crystallization of proteins would be controlled by protein’s self-interaction. To date, a three-dimensional structure of apomyoglobin by X-ray crystal analysis has not been uploaded to the Protein Data Bank, while holomyoglobin was the first success in the X-ray crystal analysis of the protein structures (Kendrew *et al.*, 1958 ▶). Unravelling how removal of heme changes the protein–protein interaction of myoglobin will be a step toward understanding key factors for the aggregation and the solubility.

## 2. Materials and methods

### 2.1. Sample preparation

Holomyoglobin from equine skeletal muscle was purchased from Sigma. Apomyoglobin was prepared according to the following methods (Teale, 1959 ▶; Hapner *et al.*, 1968 ▶). 10 mg ml^−1^ aqueous solution of myoglobin was adjusted to pH ∼1.5 with HCl at 297 K. An equal volume of ice-cooled 2-butanone was mixed with the solution, and then the organic phase containing the extracted heme was discarded. This procedure was repeated three times. The aqueous phase was dialyzed against sodium bicarbonate (0.05 g l^−1^) and then ultrapure water (Milli-Q, Millipore). The apomyoglobin solution was finally lyophilized. The solutions of holomyoglobin and apomyoglobin for the measurements were prepared by dissolving the powders into the ultrapure water. The pH of the holomyoglobin and apomyoglobin solutions were 7.5 and 6.7, respectively. The concentrations of both the proteins were determined by using an ∊_410nm_ of 1.6 × 10^5^ for holomyoglobin and ∊_280nm_ of 1.43 × 10^4^ for apomyoglobin (Goto *et al.*, 1990 ▶).

### 2.2. Small-angle X-ray measurement

SAXS was measured using a SAXSess camera (Anton Paar; Graz, Austria) with a sealed-tube X-ray source (PANalytical, The Netherlands), operating at 40 kV and 50 mA, and line collimation. X-ray intensities were recorded using an imaging plate (100 µm × 100 µm pixel size; Fuji Film, Japan), which was read using a Cyclone scanner (PerkinElmer, USA). The X-ray wavelength, λ, was 1.542 Å (Cu *K*α); the camera length was 264.5 mm. The image data were integrated into one-dimensional scattering intensities using the program *ImageJ* (developed by Wayne Rasband, National Institute of Health, Bethesda, MD, USA) with the macros in Utah SAXS Tools written by Professor David P. Goldenberg (University of Utah, USA). The scattering parameter *q* is defined as *q* = 4πsinθ/λ, where 2θ is the scattering angle of X-rays. The width of the integration area (detector slit width) was 10 mm. Protein solution and water were measured at 293 K in 1 mm-diameter glass capillaries for 1 h.

### 2.3. Calculation of a structure factor from an intermolecular potential

The SAXS intensity of a protein, *I*(*q*), is described by
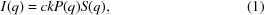
where *c* is the protein concentration, *k* is a constant, *P*(*q*) is a form factor (protein’s self-scattering) and *S*(*q*) is a structure factor (protein’s interparticle interference). The SAXS profiles of the dilute protein solutions (0.5 wt%), at the concentration of which the interparticle interferences are negligibly small, yields *kP*(*q*) by an indirect Fourier transform program *GNOM* (Svergun, 1992 ▶) involving correction of the slit smeared effect. *S*(*q*) can be connected to the intermolecular potential of a protein molecule, *V*(*r*), by solving the Ornstein–Zernike equation with an appropriate closure. In the present analysis, we used the closure obtained from a random phase approximation, under which *S*(*q*) is described by
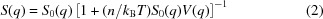
(Hansen & McDonald, 1976 ▶; Narayanan & Liu, 2003 ▶), where *S*_0_(*q*) is the structure factor of the reference system, *n* is the protein’s number density, *k*_B_ is the Boltzmann constant and *V*(*q*) is the Fourier transform of *V*(*r*). *S*_0_(*q*) has been evaluated using the empty core model (Croxton, 1975 ▶; Kelkar *et al.*, 1992 ▶). The present study employed the Derjaguin–Laudau–Verwey–Overbeek (DLVO) model potential (Verwey & Overbeek, 1948 ▶), in which *V*(*r*) is expressed as
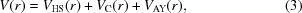
where *r* is the distance between the protein molecules. The term *V*_HS_(*r*) is the hard sphere potential given by
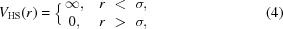
where σ is the diameter of the protein. The terms *V*_C_(*r*) and *V*_AY_(*r*) are the screened repulsive Coulomb potential and the attractive potential, respectively [here, *V*_AY_(*r*) is assumed to be a Yukawa-type potential],
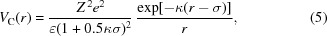


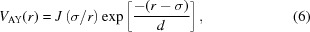
where *Z* is the net charge on the protein, *e* is the elementary charge, ∊ is the dielectric constant of the medium, κ is the reciprocal Debye–Hückel screening length, *J* is the depth of attractive potential at *r* = σ, and *d* is the range of the attractive potential. *S*(*q*) can be simulated, according to the equations described above and the given values for the parameters (*Z*, σ, *J* and *d*). In the present study, we used *Z* = 1 and σ = 41.2 Å, the values of which were from the literature (Longeville *et al.*, 2003 ▶). The procedure for the present analysis was performed by using *IGOR Pro* version 6.22A (Wavemetrics, USA).

## 3. Results and discussion

**Figure 1:**
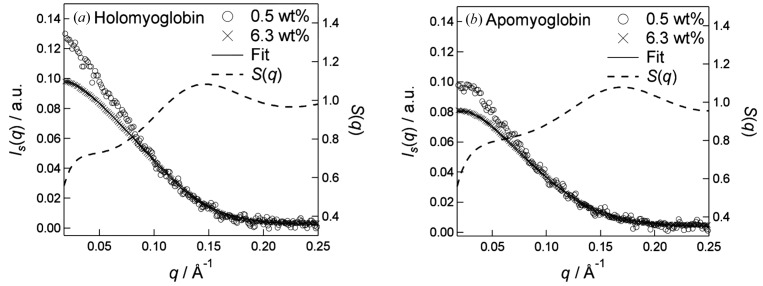
The normalized SAXS intensities of (*a*) holomyoglobin and (*b*) apomyoglobin at 6.3 wt% (crosses) and 0.5 wt% (open circles). The solid lines are theoretical fits of *I*_s_(*q*) and the dashed lines are the calculated *S*(*q*), where the *J* and *d* values (0.7 *k*_B_ *T* and 12 Å for holomyoglobin, 14 *k*_B_ *T* and 0.7 Å for apomyoglobin) were determined by the non-linear least-squares Levenberg–Marquardt algorithm.

**Figure 2:**
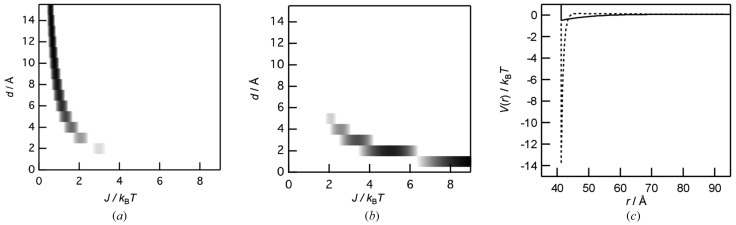
Maps of the residual sum of squares between the experimental *I*_s_(*q*) and the calculated *I*_s_(*q*) within ranges of *J* (0.01–9*k*_B_ *T*) and *d* (1–15 Å) for (*a*) holomyoglobin and (*b*) apomyoglobin. The darker portions in the map represent the values which efficiently reduce the residuals. (*c*) The protein–protein interaction potentials, *V*(*r*), are calculated from the same values of *J* and *d* in Fig. 1 ▶ (full line: holomyoglobin; dashed line: apomyoglobin).

Fig. 1 ▶ shows the observed SAXS profiles of holomyoglobin and apomyoglobin at 0.5 and 6.3 wt%, where the intensities are normalized by the protein concentrations. For both the 6.3 wt% solutions, the intensities at low scattering vectors (*q* < ∼0.1 Å^−1^) were depressed compared with that for 0.5 wt% solutions, which are due to the interparticle interference, *i.e.* the structure factor. We analysed the structure factor component in the SAXS intensities based on the DLVO model interaction potential and the equations above. A slit-smeared SAXS intensity, *I*_s_(*q*), was simulated from *I*(*q*), which is from the determined *kP*(*q*) and the theoretically derived *S*(*q*) with equation (1), and the instrumental beam profile used as a weighting function (Glatter & Kratky, 1982 ▶). To find the values of the parameters (*J* and *d*), which reproduce the experimental *I*_s_(*q*), we compared the experimental *I*_s_(*q*) with the calculated *I*_s_(*q*), varying the values of *J* (0.01–9 *k*_B_ *T*) and *d* (1–15 Å). The maps of the residual sum of squares are shown in Figs. 2(*a*) and 2(*b*) ▶. It is found that smaller *J* and larger *d* tend to reduce the residuals for holomyoglobin, while larger *J* and smaller *d* tend to reduce the residuals for apomyoglobin. The protein–protein interaction potentials, *V*(*r*), calculated with the optimal values of *J* and *d*, which give the best fits, are depicted in Fig. 2(*c*) ▶. At small *r* (*r* < ∼45 Å), the potential of apomyoglobin is more attractive than that of holomyoglobin, due to the larger *J* value. This could explain the irreversible aggregation-prone property of apomyoglobin (Fändrich *et al.*, 2003 ▶). In addition, the attractive potential of holomyoglobin is relatively small negative at small *r* (*r* < ∼45 Å), but still remains at larger *r* (*r* < ∼55 Å) due to the larger *d* value. The attractive potential allows the proteins to be close to each other, but to be separated because the magnitude is comparable with thermal energy (∼*k*_B_ *T*) at short range. This seems to underlie the highly water-soluble and less irreversible aggregation-prone properties of holomyoglobin.

## 4. Summary

In the present study we investigated the role for the heme in the self-interaction of myoglobin molecules by SAXS. From the fitting of the experimental SAXS data with the simulated SAXS intensities based on the DLVO model and the equations in the liquid theory, it is suggested that change in the SAXS profile by removal of the heme could be explained by an increase in the intermolecular attractive interaction potential at short range from the protein molecule. We remark that the potential we can obtain depends on the selected model. Therefore, we progress a study developing a method to determine the intermolecular potential of proteins from experimental structure factors without assuming any model potential by applying an integral equation theory.
